# Seasonal Variation in the Chemical Composition and Antimicrobial Activity of Volatile Oils of Three Species of *Leptospermum* (Myrtaceae) Grown in Brazil 

**DOI:** 10.3390/molecules16021181

**Published:** 2011-01-26

**Authors:** Antonio Jacinto Demuner, Luiz Claudio Almeida Barbosa, Cassia Gonçalves Magalhaes, Cleber Jose da Silva, Celia Regina Alvares Maltha, Antonio Lelis Pinheiro

**Affiliations:** 1 Departamento de Química, Universidade Federal de Vicosa, Av. P. H. Rolfs, s/n, 36570-000, Vicosa, MG, Brazil; E-Mails: ademuner@ufv.br (A.J.D.); cassiagmag@yahoo.com.br (C.G.M.); cleberbio2002@yahoo.com.br (C.J.S.); crmaltha@ufv.br (C.R.A.M.); 2 Departamento de Engenharia Florestal, Universidade Federal de Vicosa, Av. P. H. Rolfs, s/n, 36570-000, Vicosa, MG, Brazil; E-Mail: pinheiro@ufv.br (A.L.P.)

**Keywords:** Myrtaceae, *Leptospermum*, seasonal variation, essential oils, antibacterial activity

## Abstract

This study investigates the seasonal variation of three species of *Leptospermum* (Myrtaceae) grown in Brazil. The chemical composition of the volatile oils of *L. flavescens* and *L. petersonii* did not show any significant seasonal variation in the major components, while for *Leptospermum madidum* subsp. *sativum* the levels of major constituents of the volatile oils varied with the harvest season. Major fluctuations in the composition of *L. madidum* subsp. *sativum* oil included α-pinene (0–15.2%), *β*-pinene (0.3–18.5%), α-humulene (0.8–30%), 1,8-cineole (0.4–7.1%) and *E*-caryophyllene (0.4–11.9%). Levels of *β*-pinene (0.3–5.6%), terpinen-4-ol (4.7–7.2%) and nerolidol (55.1–67.6%) fluctuated seasonally in the *L. flavescens* oil. In *L.* *petersonii*, changes were noted for geranial (29.8–32.8%), citronellal (26.5–33.9%) and neral (22.7–23.5%). The activity of the volatile oils against the tested bacteria differed, depending on season the oils were obtained. In general, the volatile oils were more active against Gram-positive bacteria.

## 1. Introduction

*Leptospermum* J. R. et G. Forst. belongs to the Myrtaceae family and consists of 83 species of shrubs and trees, that are widely distributed throughout Australia and New Zealand [1]. Some species of this genus are used in traditional medicine, as exemplified by *L. flavescens*, which has been used in Malaysia to stimulate appetite and relieve stomach disorders and menstrual discomfort [2]. The volatile oils of Manuka (*Leptospermum scoparium* J.R. et G. Forst.) is of great economic value in New Zealand [3]. These oils are active against Gram-positive bacteria, including antibiotic-resistant strains [4,5,6,7,8]. In addition, *Leptospermum* honey has been reported to exhibit antimicrobial activity against *Staphylococcus* *aureus* and *Helicobacter* *pylori*, the bacteria responsible for some stomach ulcers [9,10,11]. The triketone nitisinone has been used for the treatment of tyrosinaemia type I. [12]. Furthermore, it has already been shown that triketone derivatives extracted from *L. scoparium* seeds exhibit acaricidal activity [13]. The natural β-triketone known as leptospermone have been used as a model compound for the design of commercial herbicides sulcotrione and mesotrione [14]. The ethanolic extract of *Leptospermum recurvum* showed anti-microbial activity, in addition to some anti-viral action [15]. An investigation of the chemical composition of the ethanolic extract of *L. polygalifolium* subsp. *polygalifolium* revealed the presence antimicrobial components including flavesone, leptospermone and isoleptospermone [15].

Therefore, given the important biological activities of the volatile oils produced by several species of the Myrtaceae [16], and following our previous investigations on the biological activities and composition of volatile oils from several plant species [17,18,19,20], in this work we report the results concerning the effect of the seasonal variation on the chemical composition and antimicrobial activity of the volatile oils produced by *Leptospermum flavescens, Leptospermum madidum* subsp.* sativum* and* Leptospermum petersonii*, grown in Brazil.

## 2. Results and Discussion

### 2.1. Volatile oil yields

The volatile oil yields obtained by hydrodistillation of plants collected in the dry and in the rainy seasons are shown in Table 1. For *L.* *petersonii* an increase in the yield of volatile oil in the rainy season was observed, while in *L. madidum* subsp. *sativum,* the yield of volatile oils decreased*.* The content found for the volatile oils from flowers of *L. petersonii* during the rainy season was 1.3 ± 0.1%, lower than that found for the leaves (3.7 ± 0.1%) in the same period. It is known that many factors such as season, herbivore activity, temperature and reproductive stage, the age of leaves and the growing conditions can lead to qualitative and quantitative differences in the volatile oils produced [17,21,22,23,24,25,26,27]. The largest quantity of oils found in the leaves of *L. petersonii*, in relation to the flowers (Table 1) could be associated with physiological or ecological roles in the two plant organs. In *Artemisia dracunculus*, the highest production of the essential oil is coincident with the flowering period [26].

**Table 1:** Content (% w/w) of the volatile oil of the three species of *Leptospermum* on dried matter*.*

	LM	LF	LP	FLP
**Dry season**	1.1 ± 0.1	1.5 ± 0.2	2.8 ± 0.3	-
**Rainy season**	0.7 ± 0.1	1.6 ± 0.2	3.7 ± 0.1	1.3 ± 0.1

LM = *L. madidum* ssp s*ativum*; LF = *L. flavescens*; LP = *L. petersonii*; FLP = flowers of *L. petersonii.*

### 2.2. Volatile oils quantitative analysis

Results related to seasonal variation of yield and chemical composition of essential oils of *Leptospermum* species are presented in Table 2. 

**Table 2:** Chemical composition (%) of volatile oils from *Leptospermum* species*.

		*L. madidum*		*L. flavescens*		*L. petersonii*		*Flower L. petersonii*	
Components	RI	Dry	Rainy	Dry	Rainy	Dry	Rainy	HD	SPME
*Monoterpene hydrocarbons*									
***α*-pinene**	938	**15.2 ± 0.7**	-	2.1 ± 0.6	0.2 ± 0.0	-	-	-	4.1 ± 0.2
***β*-pinene**	980	**18.5 ± 0.5**	0.3 ± 0.0	**5.6 ± 1.4**	0.3 ± 0.1	-	-	-	-
*β* -myrcene	993	0.6 ± 0.1	-	0.4 ± 0.1	-	0.6 ± 0.1	0.3 ± 0.2	0.6 ± 0.4	10.7 ± 0.5
*α*-terpinene	1017	0.3 ± 0.0	-	0.2 ± 0.0	-	-	-	-	-
*p*-cymene	1027	-	-	0.5 ± 0.1	0.5 ± 0.1	-	-	-	-
limonene	1031	0.2 ± 0.0	0.1 ± 0.0	2.6 ± 0.6	-	-	-	-	-
*β*-ocymene	1053	1.5 ± 0.5	0.1 ± 0.0	-	-	-	-	-	
*γ*-terpinene	1062	0.6 ± 0.1	0.1 ± 0.0	2.2 ± 0.3	0.5 ± 0.0	-	-	-	-
α-terpinolene	1089	0.4 ± 0.1	-	0.6 ± 0.0	-	-	-	-	-
α-cubebene	1352	0.5 ± 0.0	0.6 ± 0.2	-	-	-	-	-	0.3 ± 0.2
*Oxygenated monoterpenes*									
**1,8-cineole**	1035	**7.1 ± 0.7**	0.4 ± 0.0	2.9 ± 1.0	0.6 ± 0.1	-	-	-	-
linalool	1102	-	0.2 ± 0.1	0.1 ± 0.0	-	1.5 ± 0.0	1.6 ± 0.0	2.5 ± 0.0	1.1 ± 0.0
Rose oxide	1115	-	-	-	-	-	-	-	0.7 ± 0.3
*endo*-fenchol	1116	-	-	0.3 ± 0.1	0.2 ± 0.0	-	-	-	-
isopulegol	1150	-	-	-	-	3.4 ± 0.2	3.7 ± 0.6	4.1 ± 0.1	2.2 ± 0.3
**citronellal**	1154	-	-	-	-	**33.9 ± 1.0**	**26.5 ± 1.0**	**35.0 ± 0.5**	**27.4 ± 1.6**
borneol	1171	-	-	0.4 ± 0.5	0.3 ± 0.0	-	-	-	-
**terpinen-4-ol**	1178	-	0.7 ± 0.0	**7.2 ± 0.5**	**4.7 ± 0.3**	-	-	-	-
*α*-terpineol	1194	3.7 ± 0.0	2.2 ± 0.7	3.1 ± 0.3	2.2 ± 0.1	-	-	-	-
*β*-citronellol	1238	-	-	-	-	0.04 ± 0.0	0.1 ± 0.0	4.0 ± 0.3	**22.1 ± 3.6**
**neral**	1239	-	-	-	-	**22.7 ± 0.7**	**23.5 ± 1.5**	**16.5 ± 2.2**	**2.4 ± 0.1**
geraniol	1257	-	-	-	-	1.2 ± 0.1	1.7 ± 0.1	1.8 ± 0.0	2.0 ± 0.0
**geranial**	1269	-	-	-	-	**29.8 ± 0.4**	**32.8 ± 0.5**	**26.1 ± 0.3**	2.0 ± 0.5
methyl geranate	1330	-	-	-	-	-	-	-	1.2 ± 0.4
citronellyl acetate	1359	-	-	-	-	1.0 ± 0.0	1.0 ± 0.0	2.0 ± 0.0	0.7 ± 0.6
geranyl acetate	1387	-	-	-	-	0.5 ± 0.0	0.7 ± 0.0	0.5 ± 0.0	-
*Other Oxygenated Compounds*									
benzaldehyde	965	0.6 ± 0.0	-	-	-	-	-	-	-
( *Z*)-hex-3-en-1-yl acetate	1015	-	-	-	-	-	-	-	3.2 ± 0.1
eugenol	1362	0.3 ± 0.0	0.3 ± 0.1	-	-	0.3 ± 0.0	0.3 ± 0.0	0.9 ± 0.0	-
cinnamyl acetate	1453	-	-	-	-	-	-	0.1 ± 0.0	
*Sesquiterpene* *hydrocarbons*									
α-copaene	1380	1.0 ± 0.1	0.5 ± 0.2	0.1 ± 0.0	0.2 ± 0.0	-	-	-	0.4 ± 0.3
*β*-burbonene	1388	-	0.5 ± 0.0	-	-	-	-	-	-
*β*-elemene	1393	0.3 ± 0.0	0.2 ± 0.0	0.3 ± 0.0	0.5 ± 0.2	0.1 ± 0.0	0.4 ± 0.0	0.1 ± 0.0	3.9 ± 1.2
α-gurjunene	1412	0.6 ± 0.0	1.1 ± 0.0	-	-	-	-	-	-
***E*-caryophyllene**	1424	5.8 ± 0.0	**11.9 ± 0.1**	1.1 ± 0.2	1.2 ± 0.1	-	-	-	0.4 ± 0.1
*β*-gurjunene	1435	-	0.7 ± 0.1	-	-	-	-	-	-
aromadendrene	1443	1.5 ± 0.2	2.8 ± 0.2	-	-	-	-	-	-
**α-humulene**	1459	**10.2 ± 0.2**	**30.8 ± 1.2**	0.8 ± 0.1	1.1 ± 0.2	-	-	-	-
( *E*)-*β*-farnesene	1461	-	-	1.5 ± 0.0	0.4 ± 0.0	-	-	-	-
alloaromadendrene	1465	0.9 ± 0.1	1.2 ± 0.0	-	-	-	-	-	-
*β*-chamigrene	1470	-	-	0.5 ± 0.0	0.6 ± 0.0	-	-	-	-
*γ*-muurolene	1477	1.1 ± 0.1	0.6 ± 0.1	-	-	-	-	-	-
germacrene D	1480	1.1 ± 0.1	2.2 ± 0.1	-	-	-	-	-	-
*cis-β*-guaiene	1491	-	-	1.9 ± 0.2	-	-	-	-	-
valencene	1492	-	-	-	-	-	-	-	0.7 ± 0.6
α-selinene	1500	-	-	2.2 ± 0.3	3.0 ± 0.2	-	-	-	-
bicyclogermacrene	1501	-	-	-	-	-	-	-	1.4 ± 0.7
ledene	1501	6.3 ± 0.1	-	-	-	-	-	-	-
not identified	1508	-	10.5 ± 0.2	-	-	-	-	-	-
farnesene	1512	-	-	-	-	-	-	-	0.3 ± 0.0
*γ*-cadinene	1518	0.3 ± 0.0	-	-	-	-	-	-	-
*cis*-calamenene	1528	2.4 ± 0.0	-	-	-	-	-	-	-
δ-cadinene	1529	-	-	0.6 ± 0.1	0.8 ± 0.1	-	-	-	0.2 ± 0.0
Not identified	1535		6.6 ± 1.1						
not identified	1543		3.0 ± 1.1						
germacrene B	1562	0.7 ± 0.0	0.4 ± 0.0	-	-	-	-	-	-
*Oxygenated Sesquiterpenes*									
**nerolidol**	1570	4.8 ± 0.0	-	**55.1 ± 3.8**	**67.6 ± 0.4**	-	-	-	-
caryophyllene oxide	1577	-	-	0.1 ± 0.1	0.2 ± 0.0	-	-	-	-
not identified	1589	-	2.4 ± 0.2	-	-	-	-	-	-
viridiflorol	1595	2.0 ± 0.0	2.7 ± 0.4	-	-	-	-	-	-
TOTAL		88.2	83.1	92.4	85.1	95.0	92.6	94.7	87.4

* Average of three analyses ±SD (standard deviation). The SD = 0 corresponds to value bellow 0.05.

The volatile oils of *L. madidum* subsp. *sativum* presented as major components in the dry season *β-*pinene (18.5 ± 0.5%), *α-*pinene (15.2 ± 0.7%), *α*-humulene (10.2 ± 0.2%), 1,8-cineole (7.1 ± 0.7%), *E*-caryophyllene (5.8 ± 0.0%) and ledene (6.3 ± 0.1). Moreover, in the rainy season *E*-caryophyllene (11.9 ± 0.1%) and *α*-humulene (30.8 ± 1.2%) were the major components. Also noted were a low concentration of 1,8-cineole (0.4 ± 0.07%) and the absence of *α*-pinene. 

The volatile oils produced by *L. flavescens*, presented as a major component the sesquiterpene nerolidol in both dry and rainy seasons (55.1 ± 3.8% and 67.6 ± 0.4%, respectively). The content of other components such as *α-*pinene, *β-*pinene, *γ-*terpinene, 1,8-cineole and terpinen-4-ol showed variations in the dry and rainy season (Table 2). The high concentrations of nerolidol in the oils of *L. flavescens* grown in Viçosa suggest that this oil can be used as food flavor additives. Besides, nerolidol is a sesquiterpene that has been tested as skin penetration enhancer for the transdermal delivery of therapeutic drugs [28] and as enhancer of bacterial permeability to antibiotics and antimicrobials [29].

Citronellal (33.9 ± 1.0%; 26.5 ± 1.0%), neral (22.7 ± 0.7%; 23.5 ± 1.5%) and geranial (29.8 ± 0.4; 32.8 ± 0.5) were the main components of the volatile oils produced by leaves of *L.* *petersonii* cultivated in Viçosa in both seasons*.* The volatile oils obtained from the leaves and flowers of *L. petersonii* showed similar chemical composition. The major components identified in volatile oils produced by flowers and isolated by hydrodistillation were citronellal (35.0 ± 0.5%), neral (16.5 ± 2.2%) and geranial (26.1 ± 0.3%). The volatiles constituents identified by solid phase microextraction from the flowers of *L.* *petersonii* were citronellal (27.4 ± 1.6%) and *β*-citronellol (22.1 ± 3.6%). These compounds are pollinizators attracting and act protecting the reproductive organs and their cells germination against pathogens or damages caused by ozone [30].

As can be observed in Table 3, there was a predominance of sesquiterpenes in the volatile oil of *L. flavescens* in both seasons. In *L. madidum* subsp.* sativum*, however, there was a predominance of monoterpenes in the dry season, contrasting with the predominance of sesquiterpenes in the rainy season, while for *L. petersonii* there was predominance of monoterpenes in both periods. In the volatile oils from flowers of *L. petersonii* obtained by hydrodistillation and solid phase microextraction, there was a predominance of monoterpenes. The volatile oil of *L. flavescens* and *L. petersonii* showed predominance of oxygenated terpenes in the dry and rainy seasons. On the other hand, in the volatile oils of *L. madidum* subsp. *sativum*, there was predominance of terpene hydrocarbons in both seasons.

**Table 3:** Content (%) of different classes of terpenes in the volatile oils of species of *Leptospermum.*

	LM		LF		LP		LP flower	
	Dry	Rainy	Dry	Rainy	Dry	Rainy	HD	SPME
**Monoterpene hydrocarbon**	37.8	1.2	14.2	1.5	0.6	0.3	0.6	15.1
**Oxygenated monoterpenes**	11.7	3.8	14.0	8.0	94.3	91.9	94.0	65.0
**Sesquiterpene hydrocarbon**	31.9	73.0	9.0	7.8	0.1	0.4	0.1	7.3
**Sesquiterpene Oxygenated**	6.8	5.1	55.2	67.8	-	-	-	-
**Hydrocarbon/ Oxygenated**	69.7/18.5	74.2/8.9	23.2/69.2	9.3/75.8	0.7/94.3	0.7/91.9	0.7/94.0	22.4/65.0

LM = *L. madidum* ssp s*ativum*; LF = *L. flavescens*; LP = *L. petersonii*.

### 2.3. Antibacterial activity

The results of the antimicrobial biological assays of the volatile oils from *Leptospermum* species by the agar diffusion method are shown in Table 4. In general the volatile oils were more active against Gram-positive bacteria. With the exception of volatile oil of *L. petersonii*, the volatile oils evaluated in this screening did not cause inhibition of Gram-negative *Escherichia coli*. The volatile oils were effective against Gram-positive *Bacillus cereus* and *Staphylococcus aureus*. The volatile oil of *L*. *flavescens* exhibited activity against *S. aureus* and*B. cereus.* The activity against Gram-positive bacteria can be related to the high concentration of nerolidol present in the volatile oils of this species (55.1 ± 3.8% and 67.6 ± 0.4%, dry and rainy seasons respectively). These results are similar to those found for antibacterial activity of volatile oil of *Momordica charantia* which has high levels of nerolidol [31]. This activity suggests that the damage was caused to the cellular membrane [32]. Kubo *et al*. [33] proposed a relationship between antibacterial activity and the structure of aliphatic alcohols such as nerolidol. They suggested that maximum activity against *S. aureus* might be dependent on the number of carbon atoms in the hydrophobic chain from hydrophilic hydroxyl group, which should be less than twelve but as close to twelve as possible. The corresponding chain in nerolidol has ten carbon atoms. The nature of the functional groups and configuration of double bonds probably affected the activity even though aliphatic chain length might be a dominant indicator of antibacterial activity. However, nerolidol has been inactive against E. *coli* [34]. Apparently, the maximum activity of the carbon chain lengths differed between the microorganisms, due to differences in their cell-envelope structures [35].

The volatile oil of *L. madidum* subsp. *sativum* exhibited activity against *S. aureus* in both periods, being more effective against *B. cereus* in dry period (Table 4). In this period, the volatile oil of that species presents higher concentrations of *α*-pinene (15.2 ± 0.7%), *β*-pinene (18.5 ± 0.5%) and 1,8-cineole (7.1 ± 0.7%). It has been demonstrated that *α*-pinene and *β*-pinene are able to destroy cellular integrity, and thereby, inhibit respiration and ion transport processes. They also increase the membrane permeability in yeast cells and isolated mitochondria [36]. However the volatile oils of the *L. flavescens* and *Leptospermum madidum* subsp. *sativum* did not show activity against *E. coli*. 

**Table 4:** Antibacterial activities of the volatile oils from Leptospermum species.

		Inhibition zone diameter (mm)		
		Gram-positive		Gram-negative
Species	Season	*B. cereus*	*S. aureus*	*E. coli*
*L. madidum sativum*	Dry	1.87 ± 0.12cA	1.03 ± 0.03bB	0.60 ± 0.00cC
	Rainy	1.20 ± 0.00*dA	1.07 ± 0.07bB	0.60 ± 0.00cB
*L. flavescens*	Dry	1.12 ± 0.05dA	0.87 ± 0.09bB	0.60 ± 0.00cC
	Rainy	1.10 ± 0.10dA	0.90 ± 0.06bA	0.60 ± 0.00cB
*L. petersonii*	Dry	4.17 ± 0.27aA	2.07 ± 0.17aB	0.83 ± 0.07bC
	Rainy	3.77 ± 0.14bA	2.10 ± 0.10aB	1.13 ± 0.09aC
Control		0.60 ± 0.00eA^b^	0.60 ± 0.00cA	0.60 ± 0.00cA

Means followed by same capital letter in the lines and small letter in the columns are not different for the Scott-Knott's test at P ≤ 0.05. *SD value below 0.005 was rounded to 0.00.

The volatile oils of *L. petersonii* were active against all microorganisms tested. In both the dry seasons and rainy season, the volatile oil of this species showed high levels of geranial (29.8 ± 0.4% and 32.8 ± 0.5%, respectively) and citronellal (33.9 ± 1.0% and 26.5 ± 1.0%, respectively). *B. cereus* was extremely sensitive to the oil of that species in the dry period. For *S. aureus*, the inhibition of the growth was same in the dry period and after the occurrence of rains. Aldehydes may play an important role in the observed antimicrobial activity of plant aldehyde-containing material. Their action, very likely due to an alteration in the function of membrane-associated proteins, seems to be exerted mainly at the cell surface [37,38]. However, the ability to penetrate the outer layer of cells can help to explain the antimicrobial activity of some aldehydes [38]. Kubo *et al*. [33] have suggested that the carbon chain length influences the disorder in the lipidic bilayer. Similarly, the antimicrobial activity of a series of long-chain alcohols was shown to depend on the alkyl chain length. 

## 3. Experimental

### 3.1. Plant material

Leaves of *Leptospermum madidum* subsp. *sativum* A.R. Bean*, Leptospermum flavescens* Smith *e Leptospermum petersonii* F.M. Bailey were collected separately in September and December 2007, from plants grown in the arboretum of the Forest Engineering Department, Dendrology Sector, at the Federal University of Viçosa (UFV), Minas Gerais State, Brazil. The materials were identified, herborized and a voucher specimen of each plant has been deposited in the VIC Herbarium (registration numbers are 21524 and 21585) of the Plant Biology Department, Federal University of Viçosa (UFV). The flowers of *L. petersonii* were collected in December 2007 for analysis of the volatiles and volatile oil (registration number is 21586).

### 3.2. Hydrodistillation

Leaves were collected separately in a completely randomized way from the trees under investigation. Each sample was subdivided into three portions of 50 g each, chopped and then subjected to a three hours hydrodistillation in a Clevenger-type apparatus. The resulting oils were weighed and the reported yields were calculated in relation to the dry matter. The oils were stored under nitrogen atmosphere, at -4 °C, until they were analyzed by gas chromatography and mass spectrometry (GC-MS). Leaf dry matter mass was calculated by drying each sample (1 g, held at 120 ± 2 °C until constant mass) according to published methods [39]. Each determination was carried out in triplicate.

### 3.3. Headspace solid phase microextraction (HS-SPME) and Gas chromatography-Mass spectrometry GC-MS analysis

For HS-SPME sampling, a 75 µm polydimethylsiloxane (PDMS) fiber was used. Prior to use, the SPME fiber was conditioned in the hot GC injection port at 120 °C for 1 h in order to remove contaminants. Approximately 1 g of flowers collected at 10 am was placed in a 30 mL vial and the vial was sealed with aluminum seal and rubber septum. The vial was left to equilibrate at 40 °C for at least 40 min before HS-SPME. After adsorption, the SPME fiber was removed from the sample vial and immediately inserted into the injection port of the GC–MS system where the thermal desorption was performed at 240 °C for 1 min. The GC-MS unit (model GCMS-QP5050A, from Shimadzu, Japan) was equipped with a DB-5 fused silica column (30 m × 0.25 mm i.d., film thickness 0.25 μm) and interfaced with an ion trap detector. Injector temperature was 220 °C; transfer line temperature, 240 °C; ion trap temperature, 220 °C; carrier gas, He at a flow rate of 1.8 mL min^-1^; detector temperature 240 °C; column temperature was programmed to start at 55 °C (isothermal for 2 min), with an increase of 3 °C min^-1^, to 240 °C, isothermal at 240 °C for 30 min; splitless; column pressure of 118 kPa; ionization energy, 70 eV; scan range, 29–450 u; scan time, 1 s. The identity of each component was assigned by comparison of their retention indexes (RRI), relative to a standard alkane series (C_9_-C_27_) and also by comparison of its mass spectrum with either reference data from the equipment database (Wiley 330.000) or from the literature [40]. For the oils extracted the GC-MS analyses were carried out under the same conditions described above. The samples were dissolved in CH_2_Cl_2 _(1% w/v) and 1.0 μL was injected using a split ratio of 1:10. 

### 3.4. Gas chromatography analysis of the volatile oils

GC analyses were carried out with a GC-17A Series instrument (Shimadzu, Japan) equipped with a flame ionization detector (FID). Chromatographic conditions were as follows: fused silica capillary column (30 m × 0.25 mm) with a DB-5 bonded phase (0.25 μm film thickness); carrier gas, N_2_ at a flow rate of 1.8 mL min^-1^; injector temperature 220 °C, detector temperature 240 °C; column temperature was programmed to start at 40 °C (isothermal for 2 min), with an increase of 3 °C min^-1^, to 240 °C, isothermal at 240 °C for 20 min; injection of 1.0 μL (1% w/v in CH_2_Cl_2_); split ratio 1:10; column pressure of 118 kPa. The analyses were carried out in triplicate and the amount of each compound was expressed as a relative percentage of the total area of the chromatograms.

### 3.5. Bacterial strains

Bacterial strains were obtained from the collections of the Department of Microbiology, Federal University of Viçosa, Viçosa, Minas Gerais state, Brazil. Microorganisms used were Gram-positive *Staphylococcus aureus* (ATCC 25923) and *Bacillus cereus,* Ribotype 1 222-173-S4 isolated from equipment surfaces post-pasteurization [41]; Gram-negative *Escherichia coli* (ATCC 11229). Organisms were maintained in nutrient agar (Sigma) at 37 °C. Overnight cultures were prepared in Brain Heart Infusion Broth (Himedia) and adjusted to approximately 10^8^ CFU mL^-1^.

### 3.6. Antibacterial screening

The agar disc diffusion method was employed to determine the antimicrobial activity of the volatile oils, as previously described [42]. Briefly, a suspension of the tested microorganism (2 × 10^8^ CFU mL^-1^) was spread on Petri plates with Mueller Hinton agar. Filter paper discs (6 mm diameter) were individually impregnated with 10 µl of the volatile oils and placed on the inoculated plates. The plates were incubated for 48 hours at 37 °C in the cases of *S. aureus* and *E. coli* and at 32 °C for *B. cereus*. The diameters of the inhibition zones were measured using a paquimeter and expressed in millimeters. Sterile water served as negative control. Each test was performed in three triplicates and repeated three times. The results were analyzed by ANOVA and Scott-Knott’s multiple-range tests at P ≤ 0.05 by using the software GENES (Genetics and Statistical Analysis. Version 2007.0.0 - Federal University of Viçosa, Viçosa – MG, Brazil).

## 4. Conclusions

In summary, the seasonal variation indicate similar chemical profiles for of *L. flavescens* and *L. petersonii* and different chemical profile in *Leptospermum madidum* subsp. *sativum* with respect to seasonal fluctuation in the levels of major constituents of the volatile oils. This study shows that the volatile oils of *Leptospermum* species showed better antimicrobial activity against the Gram-positive bacteria when compared to the Gram-negative bacteria. 
